# Salvage technique for covered metal stent migration during endoscopic reintervention after endoscopic ultrasound-guided hepaticogastrostomy

**DOI:** 10.1055/a-2228-4400

**Published:** 2024-01-17

**Authors:** Yuki Ikeda, Daichi Watanabe, Ginji Oomori, Shota Yamada, Toshinori Okuda, Shinya Minami

**Affiliations:** 1 36937 Department of Gastroenterology, Medical Corporation Oji General Hospital, Tomakomai, Japan

Endoscopic ultrasound (EUS)-guided hepaticogastrostomy (EUS-HGS) is an alternative drainage method for malignant biliary obstruction (MBO) when endoscopic retrograde cholangiopancreatography (ERCP) has failed 1 . A partially covered self-expandable metal stent (PCSEMS) is often used for EUS-HGS, but it cannot be removed. A new metal or plastic stent is therefore placed as endoscopic reintervention following EUS-HGS 2 3 4 ; however, troubleshooting after endoscopic reintervention remains problematic because of a paucity of reported cases.

An 81-year-old woman who had previously undergone ERCP for MBO due to pancreatic cancer presented with recurrent biliary obstruction and duodenal stricture. An EUS-HGS using a PCSEMS (EGIS biliary stent, double-covered, 8 mm × 12 cm; S&G Biotech Inc., Yongin, South Korea) and duodenal stent placement were successfully performed. After 5 months, the patient underwent endoscopic reintervention for recurrent biliary obstruction. An additional fully covered SEMS (FCSEMS; HANAROSTENT benefit, 8 mm × 8 cm; Boston Scientific Co., Tokyo, Japan) was deployed through the stent mesh of the EUS-HGS PCSEMS because of the difficulty removing the PCSEMS, along with placement of an antegrade stent across the MBO ( Fig. 1 ). A second endoscopic reintervention was required for SEMS occlusion, during which new plastic stents (Through & Pass Type-IT; Gadelius Medical, Tokyo, Japan) were placed through the distal end of the EUS-HGS SEMS after the stent mesh had been broken using argon plasma coagulation ( Fig. 2 ).

**Fig. 1:**
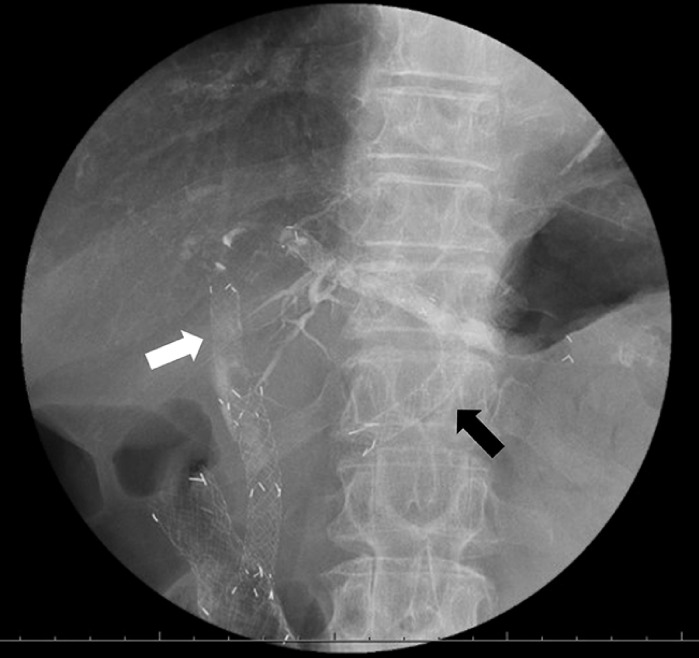
Fluoroscopic image showing endoscopic reintervention for recurrent biliary obstruction. Because the partially covered self-expandable metal stent (PCSEMS) inserted for endoscopic ultrasound-guided hepatogastrostomy (EUS-HGS) was not removed, an additional SEMS (black arrow) was deployed through the stent mesh of the EUS-HGS PCSEMS, with an additional antegrade stent placed across the malignant biliary obstruction (white arrow).

**Fig. 2:**
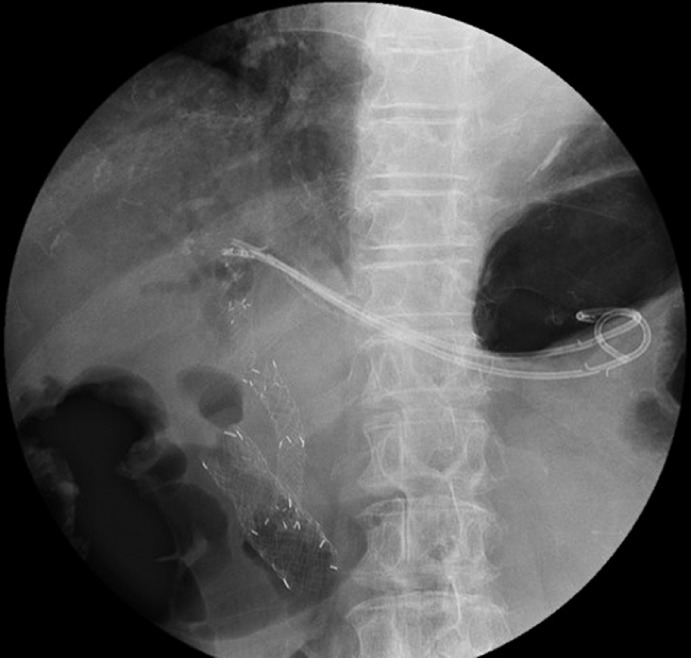
During a second endoscopic reintervention for self-expandable metal stent occlusion, new plastic stents were placed through the distal end of the endoscopic ultrasound-guided hepatogastrostomy (EUS-HGS) self-expandable metal stent (SEMS), after the stent mesh had been broken by argon plasma coagulation, because of the difficulty removing the EUS-HGS SEMS.

After 2 months, the patient developed acute cholangitis due to migration of the EUS-HGS SEMS placed during the first endoscopic reintervention and cholecystitis due to the antegrade SEMS. After the plastic stents had been removed, grasping forceps (Rat Tooth; Olympus, Tokyo, Japan) were inserted via the EUS-HGS SEMS. The migrated SEMS was grabbed ( Fig. 3 **a** ) and removed. Additionally, the antegrade SEMS was firmly grasped and gradually removed via the EUS-HGS route ( Fig. 3 **b** ; Video 1 ). The successful removal of the two SEMSs was followed by the insertion of new plastic stents and the patient’s cholangitis and cholecystitis subsequently improved.

**Fig. 3:**
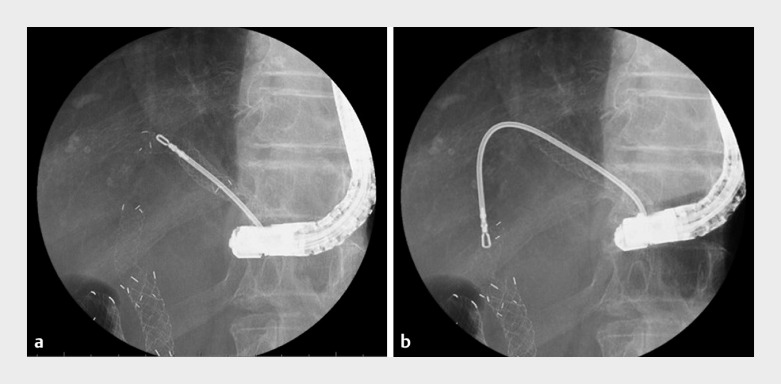
Fluoroscopic images showing: **a** the migrated self-expandable metal stent (SEMS) in the left intrahepatic bile duct being grasped with grasping forceps and removed via the hepaticogastrostomy; **b** the antegrade SEMS in the common bile duct being grasped with grasping forceps and gradually removed via the hepaticogastrostomy.

Endoscopic removal of a migrated self-expandable metal stent (SEMS) and antegrade SEMS using grasping forceps via the hepaticogastrostomy route.Video 1

This technique demonstrates successful troubleshooting of endoscopic reintervention after EUS-HGS.

Endoscopy_UCTN_Code_TTT_1AS_2AD

## Notes

Endoscopy E-Videos https://eref.thieme.de/e-videos
*E-Videos* is an open access online section of the journal *Endoscopy* , reporting on interesting cases and new techniques in gastroenterological endoscopy. All papers include a high-quality video and are published with a Creative Commons CC-BY license. Endoscopy E-Videos qualify for HINARI discounts and waivers and eligibility is automatically checked during the submission process. We grant 100% waivers to articles whose corresponding authors are based in Group A countries and 50% waivers to those who are based in Group B countries as classified by Research4Life (see: https://www.research4life.org/access/eligibility/ ). This section has its own submission website at https://mc.manuscriptcentral.com/e-videos .
